# Supplementary material for: Bursts of Genomic Instability Potentiate Phenotypic and Genomic Diversification in Saccharomyces cerevisiae
Source: Front Genet. 2022 Jun 17;13:912851. doi: 10.3389/fgene.2022.912851 (PMC9247159; doi:10.3389/fgene.2022.912851)
Supplement: Supplementary file 1 [file DataSheet2.PDF]

Table S2. Karyotypic analysis of variant genomes. A/B delineate each homolog in a pair. Bold cells indicate aneuploidies.

Isolate Name	Variant Class	Chr1		Chr2		Chr3		Chr4		Chr5		Chr6		Chr7		Chr8		Chr9		Chr10		Chr11		Chr12		Chr13		Chr14		Chr15		Chr16		total # *de novo* SVs *Chr1*b* excluded	Non-CCNA SVs
		A	B	A	B	A	B	A	B	A	B	A	B	A	B	A	B	A	B	A	B	A	B	A	B	A	B	A	B	A	B	A	B		
1_SV	smooth	1	-1	1	-1	1	-1	1	-1	1	-1	1	-1	1	-1	1	-1	1	-1	1	-1	1	-1	1	-1	1	-1	1	-1	1	-1	1	-1	1	Deletion of Chr5b (437267-Chr5RTel), Amplification of Chr13a (502144-Chr13RTel).
102_SV	smooth	1	-1	1	-1	1	-1	2	1	1	-1	1	-1	2	1	1	-1	1	-1	1	-1	1	-1	1	-1	1	-1	1	-1	1	-1	1	-1	2	
15_SV	smooth	1	-1	1	-1	1	-1	1	-1	1	-1	1	-1	1	-1	1	-1	1	-1	2	1	1	-1	1	-1	1	-1	1	-1	1	2	1	-1	2	
17_SV	smooth	1	-1	1	-1	1	-1	2	1	1	-1	1	-1	2	1	1	-1	1	-1	1	-1	1	-1	1	-1	1	-1	1	-1	1	-1	1	-1	2	
18_SV	smooth	1	-1	1	-1	1	0	1	-1	1	-1	1	-1	1	-1	1	-1	1	-1	1	-1	1	-1	1	-1	1	-1	1	-1	1	-1	1	-1	1	
19_SV	smooth	1	-1	1	-1	1	-1	1	-1	1	-1	1	-1	1	-1	1	-1	1	-1	1	-1	1	-1	1	-1	1	-1	1	-1	2	1	1	-1	2	LOH Chr13a (324469-326492)
2_SV	smooth	1	0	1	-1	1	-1	1	-1	1	-1	1	-1	1	-1	1	-1	1	-1	1	-1	1	-1	1	-1	1	-1	1	-1	2	1	1	-1	2	Reciprocal crossover Chr15 (Chr15b (Ltel-325671) Chr15a (325671-Rtel)
20_SV	smooth	1	-1	2	0	1	2	1	-1	2	1	1	2	2	1	1	-1	2	1	1	2	1	2	2	1	2	1	2	1	2	2	2	2	16	
22_CV	complex	1	-1	1	-1	1	-1	1	-1	1	-1	1	-1	1	-1	1	-1	1	-1	1	-1	1	-1	1	-1	1	-1	1	-1	1	-1	1	-1	1	Deletion of Chr2a (352193-Chr2RTel), Deletion of Chr14a (Chr14LTel-631061)
23_CV	complex	1	-1	1	-1	1	-1	1	-1	1	-1	1	-1	1	-1	1	-1	1	-1	1	2	1	-1	1	-1	1	2	1	-1	1	-1	1	-1	2	
24_CV	complex	1	-1	1	-1	1	-1	2	1	1	-1	1	-1	1	-1	1	-1	1	-1	1	-1	1	-1	1	-1	2	1	1	-1	1	-1	1	-1	2	
25_CV	complex	1	-1	1	-1	1	-1	1	-1	1	-1	1	-1	1	-1	1	-1	1	2	1	-1	1	-1	1	-1	1	2	1	-1	2	1	1	-1	4	reciprocal crossover (Chr9b (Ltel-336504) Chr9a (336998-Rtel)
26_CV	complex	1	-1	1	-1	1	-1	1	-1	1	-1	1	-1	1	-1	1	-1	1	-1	1	-1	1	-1	1	-1	1	2	1	-1	1	-1	1	-1	1	
27_CV	complex	1	-1	1	-1	1	-1	1	-1	1	-1	1	-1	1	-1	1	-1	1	-1	1	-1	1	-1	1	-1	1	-1	1	-1	1	-1	1	-1	1	Amplification of Chr4b (979209-Chr4RTel), Amplification of Chr13b (Chr13LTel-370517).
28_CV	complex	0	1	1	-1	1	-1	1	-1	1	-1	1	-1	1	-1	1	-1	1	-1	2	1	1	-1	1	-1	1	-1	1	-1	1	-1	1	-1	2	
29_SV	smooth	1	-1	1	-1	1	-1	1	2	1	-1	1	-1	1	2	1	-1	1	-1	2	2	1	-1	1	-1	1	-1	1	-1	1	2	1	-1	5	
3_SV	smooth	1	0	1	-1	1	-1	1	-1	1	-1	1	-1	1	-1	0	1	1	-1	1	1	1	-1	1	-1	1	-1	1	-1	1	-1	1	-1	1	
30_SV	smooth	1	-1	1	-1	1	-1	1	2	1	-1	1	-1	1	-1	1	-1	0	1	1	-1	1	-1	1	-1	1	-1	1	-1	1	-1	1	-1	2	
31_SV	smooth	1	-1	1	-1	1	-1	1	-1	1	-1	1	-1	1	-1	1	-1	1	-1	1	-1	1	-1	1	-1	1	-1	1	-1	2	1	1	-1	1	
32_SV	smooth	1	-1	1	-1	1	-1	1	-1	1	-1	1	2	1	-1	1	-1	1	-1	1	-1	1	-1	1	-1	1	2	1	-1	1	2	1	-1	3	
33_SV	smooth	1	-1	1	-1	1	-1	1	-1	0	1	1	-1	1	-1	1	-1	1	-1	1	-1	1	-1	1	-1	1	-1	1	-1	1	-1	1	-1	1	
34_SV	smooth	1	-1	2	1	1	-1	1	2	1	-1	1	-1	1	-1	1	-1	1	2	1	2	1	-1	1	-1	1	-1	1	2	1	-1	2	1	6	
35_CV	complex	1	-1	1	-1	1	-1	1	-1	1	-1	1	-1	1	-1	1	-1	1	-1	1	-1	1	-1	1	-1	2	1	1	-1	1	-1	1	-1	2	LOH Chr14b (203938-203424), LOH Chr13a (213-
36_CV	complex	1	0	1	-1	1	-1	1	-1	1	-1	1	0	1	-1	1	-1	1	-1	1	0	1	-1	1	-1	1	-1	1	-1	1	-1	0	1	3	
37_CV	complex	1	0	1	-1	1	-1	1	-1	1	-1	1	-1	1	-1	1	-1	1	-1	1	-1	1	-1	1	-1	2	1	1	-1	1	-1	1	-1	1	
38_CV	complex	1	-1	1	-1	1	-1	1	-1	1	-1	1	-1	1	-1	1	-1	1	-1	1	-1	1	-1	1	-1	1	2	1	-1	1	-1	1	-1	1	
39_CV	complex	1	-1	1	-1	1	-1	1	-1	1	-1	1	-1	1	-1	1	-1	1	-1	1	-1	1	-1	1	-1	1	2	1	-1	1	-1	1	-1	1	
4_SV	smooth	1	0	1	-1	1	-1	1	-1	1	-1	1	-1	1	-1	1	-1	1	-1	0	2	1	-1	1	-1	1	-1	1	-1	1	-1	1	-1	2	
40_CV	complex	2	2	1	2	1	-1	1	-1	1	-1	1	1	1	2	2	1	2	1	2	1	1	-1	1	-1	1	-1	1	-1	1	-1	2	1	9	Recombination between Chr1a/Chr1b to generate a new linear molecule.
41_CV	complex	1	2	2	1	1	-1	1	-1	1	-1	1	-1	1	-1	1	-1	1	-1	1	-1	1	-1	1	-1	2	1	1	-1	1	-1	1	2	4	
42_CV	complex	1	-1	1	-1	1	-1	1	-1	1	-1	1	-1	1	-1	1	-1	1	-1	1	-1	1	-1	1	-1	2	1	1	-1	1	-1	2	1	3	LOH Chr8b (64205-72704)
43_SV	smooth	1	-1	1	-1	1	-1	1	-1	1	-1	1	-1	1	-1	1	-1	1	-1	1	-1	1	-1	1	-1	1	-1	1	-1	1	2	1	-1	1	
44_SV	smooth	1	-1	2	1	1	-1	1	-1	1	-1	1	-1	1	-1	1	-1	2	1	1	-1	1	-1	1	-1	1	-1	1	-1	1	2	1	-1	3	
45_CV	complex	1	-1	1	-1	1	-1	1	-1	1	-1	1	-1	1	-1	1	-1	1	-1	1	-1	1	-1	1	-1	2	1	1	-1	1	-1	1	-1	1	
46_CV	complex	1	-1	1	-1	1	-1	1	-1	1	-1	1	-1	1	-1	1	-1	1	-1	1	-1	1	-1	1	-1	1	-1	1	-1	1	-1	1	-1	1	Amplification of Chr13 (Chr13LTel-480490), Amplification of Chr16 (745828-Chr16RTel)
47_CV	complex	1	-1	1	-1	1	-1	1	-1	1	-1	1	-1	1	2	1	-1	1	-1	2	1	1	-1	1	-1	1	-1	1	-1	1	-1	1	-1	2	
48_CV	complex	1	-1	1	-1	1	-1	1	-1	1	-1	1	-1	1	-1	1	-1	1	-1	1	-1	1	-1	1	-1	2	1	1	-1	1	-1	1	-1	1	
49_CV	complex	1	-1	1	-1	1	-1	1	-1	1	-1	1	-1	1	-1	2	1	1	-1	1	-1	1	-1	1	-1	1	-1	1	-1	1	-1	1	-1	3	LOH Chr4:55515-65727 (Chr4b), Chr4:532053-559335 (chr4a)
5_SV	smooth	1	-1	1	-1	1	-1	2	2	1	-1	1	-1	1	-1	1	-1	1	-1	1	2	1	-1	1	2	2	2	1	-1	1	-1	1	-1	6	
50_SV	smooth	1	-1	1	-1	1	-1	1	-1	1	-1	1	-1	1	-1	1	-1	1	-1	1	-1	1	-1	1	-1	1	-1	1	-1	2	1	1	-1	1	
51_SV	smooth	1	-1	1	-1	1	-1	1	-1	1	-1	1	-1	1	-1	1	-1	1	-1	1	-1	1	-1	1	-1	1	-1	1	-1	1	2	1	-1	1	
52_SV	smooth	1	-1	1	2	1	-1	1	-1	1	-1	1	2	2	1	1	-1	2	1	1	-1	1	-1	1	2	1	-1	1	-1	1	2	1	-1	6	
55_CV	complex	1	-1	1	-1	1	-1	1	-1	1	-1	1	-1	1	-1	1	-1	1	-1	1	-1	1	-1	1	-1	2	1	1	-1	1	-1	1	-1	1	
57_WT	wild type	1	-1	1	-1	1	-1	1	-1	1	-1	1	-1	1	-1	1	-1	1	-1	1	-1	1	-1	1	-1	1	-1	1	-1	1	-1	1	-1	0	
58_WT	wild type	1	-1	1	-1	1	-1	1	-1	1	-1	1	-1	1	-1	1	-1	1	-1	1	-1	1	-1	1	-1	1	-1	1	-1	1	-1	1	-1	0	
59_WT	wild type	1	-1	1	-1	1	-1	1	-1	1	-1	1	-1	1	-1	1	-1	1	-1	1	-1	1	-1	1	-1	1	-1	1	-1	1	-1	1	-1	0	
6_SV	smooth	1	-1	1	-1	1	-1	1	-1	1	0	1	-1	1	-1	1	-1	1	-1	1	-1	1	-1	1	-1	1	-1	1	-1	1	-1	1	-1	1	
60_WT	wild type	1	-1	1	-1	1	-1	1	-1	1	-1	1	-1	1	-1	1	-1	1	-1	1	-1	1	-1	1	-1	1	-1	1	-1	1	-1	1	-1	0	
61_WT	wild type	1	-1	1	-1	1	-1	1	-1	1	-1	1	-1	1	-1	1	-1	1	-1	1	-1	1	-1	1	-1	1	-1	1	-1	1	-1	1	-1	0	
62_WT	wild type	1	-1	1	-1	1	-1	1	-1	1	-1	1	-1	1	-1	1	-1	1	-1	1	-1	1	-1	1	-1	1	-1	1	-1	1	-1	1	-1	0	
63_WT	wild type	1	0	1	-1	1	-1	1	-1	1	-1	1	-1	1	-1	1	-1	1	-1	1	-1	1	-1	1	-1	1	-1	1	-1	1	-1	1	-1	0	
64_WT	wild type	1	0	1	-1	1	-1	1	-1	1	-1	1	-1	1	-1	1	-1	1	-1	1	-1	1	-1	1	-1	1	-1	1	-1	1	-1	1	-1	0	
65_WT	wild type	1	-1	1	-1	1	-1	1	-1	1	-1	1	-1	1	-1	1	-1	1	-1	1	-1	1	-1	1	-1	1	-1	1	-1	1	-1	1	-1	0	
66_WT	wild type	1	0	1	-1	1	-1	1	-1	1	-1	1	-1	1	-1	1	-1	1	-1	1	-1	1	-1	1	-1	1	-1	1	-1	1	-1	1	-1	0	
77_WT	wild type	1	-1	1	-1	1	-1	1	-1	1	-1	1	-1	1	-1	1	-1	1	-1	1	-1	1	-1	1	-1	1	-1	1	-1	1	-1	1	-1	0	
78_WT	wild type	1	-1	1	-1	1	-1	1	-1	1	-1	1	-1	1	-1	1	-1	1	-1	1	-1	1	-1	1	-1	1	-1	1	-1	1	-1	1	-1	0	
79_WT	wild type	1	-1	1	-1	1	-1	1	-1	1	-1	1	-1	1	-1	1	-1	1	-1	1	-1	1	-1	1	-1	1	-1	1	-1	1	-1	1	-1	0	
80_WT	wild type	1	-1	1	-1	1	-1	1	-1	1	-1	1	-1	1	-1	1	-1	1	-1	1	-1	1	-1	1	-1	1	-1	1	-1	1	-1	1	-1	0	
81_WT	wild type	1	0	1	-1	1	-1	1	-1	1	-1	1	-1	1	-1	1	-1	1	-1	1	-1	1	-1	1	-1	1	-1	1	-1	1	-1	1	-1	0	
82_WT	wild type	1	-1	1	-1	1	-1	1	-1	1	-1	1	-1	1	-1	1	-1	1	-1	1	-1	1	-1	1	-1	1	-1	1	-1	1	-1	1	-1	0	
83_WT	wild type	1	-1	1	-1	1	-1	1	-1	1	-1	1	-1	1	-1	1	-1	1	-1	1	-1	1	-1	1	-1	1	-1	1	-1	1	-1	1	-1	0	
84_WT	wild type	1	-1	1	-1	1	-1	1	-1	1	-1	1	-1	1	-1	1	-1	1	-1	1	-1	1	-1	1	-1	1	-1	1	-1	1	-1	1	-1	0	
85_WT	wild type	1	-1	1	-1	1	-1	1	-1	1	-1	1	-1	1	-1	1	-1	1	-1	1	-1	1	-1	1	-1	1	-1	1	-1	1	-1	1	-1	0	
86_WT	wild type	1	-1	1	-1	1	-1	1	-1	1	-1	1	-1	1	-1	1	-1	1	-1	1	-1	1	-1	1	-1	1	-1	1	-1	1	-1	1	-1	0	
97_SV	smooth	1	0	1	-1	1	-1	1	-1	1	0	1	-1	1	-1	1	-1	1	-1	1	-1	1	-1	1	-1	1	-1	1	-1	1	-1	1	-1	1	
98_SV	smooth	1	-1	1	-1	1	-1	1	-1	1	-1	1	-1	1	-1	1	0	1	-1	1	-1	1	-1	1	-1	1	-1	1	-1	1	-1	1	-1	2	LOH Chr13b (771650-775245)
99_SV	smooth	1	-1	1	-1	1	-1	1	-1	1	-1	1	-1	1	-1	1	-1	0	1	1	-1	1	-1	1	-1	1	-1	1	-1	1	-1	1	-1	1	
