# Supplementary material for: Bursts of Genomic Instability Potentiate Phenotypic and Genomic Diversification in Saccharomyces cerevisiae
Source: Front Genet. 2022 Jun 17;13:912851. doi: 10.3389/fgene.2022.912851 (PMC9247159; doi:10.3389/fgene.2022.912851)
Supplement: Supplementary file 2 [file DataSheet4.PDF]

Table S4. Karyotypic analysis of mutation accumulation lines derived from variant clones. A/B delineate each homolog in a pair. Bold cells indicate structural variations. Blue cells indicate de novo SVs gained during MA experiment.

Isolate Name	Variant Class	Chr1		Chr2		Chr3		Chr4		Chr5		Chr6		Chr7		Chr8		Chr9		Chr10		Chr11		Chr12		Chr13		Chr14		Chr15		Chr16		SVs in parent	# *de novo* CCNAs	# de novo CN-LOH	total # *de novo* SVs	Non-CCNA SVs
		A	B	A	B	A	B	A	B	A	B	A	B	A	B	A	B	A	B	A	B	A	B	A	B	A	B	A	B	A	B	A	B					
YJM311-1WT_MA	WT	1	1	1	1	1	1	1	1	1	1	1	1	1	1	1	1	1	1	1	1	1	1	1	1	1	1	1	1	1	1	1	1	0	0	0	0	
YJM311-2WT_MA	WT	1	1	1	1	1	1	1	1	1	1	1	1	1	1	1	1	1	1	1	1	1	1	1	1	1	1	1	1	1	1	1	1	0	0	0	0	
YJM311-3WT_MA	WT	1	1	1	1	1	1	1	1	1	1	1	1	1	1	1	1	1	1	1	1	1	1	1	1	1	1	1	1	1	1	1	1	0	0	0	0	
YJM311-4WT_MA	WT	1	1	1	1	1	1	1	1	1	1	1	1	1	1	1	1	1	1	1	1	1	1	1	1	1	1	1	1	1	1	1	1	0	0	1	1	LOH Chr2:294160-Rtel (Chr2a)
YJM311-5WT_MA	WT	1	1	1	1	1	1	1	1	1	1	1	1	1	1	1	1	1	1	1	1	1	1	1	1	1	1	1	1	1	1	1	1	0	0	2	2	LOH Chr9:267263-273958 (Chr9b), LOH Chr16:608450-Rtel (Chr16a)
YJM311-6WT_MA	WT	1	1	1	1	1	1	1	1	1	1	1	1	1	1	1	1	1	1	1	1	1	1	1	1	1	1	1	1	1	1	1	1	0	0	1	1	LOH Chr9:Ltel-219169 (Chr9b)
YJM311-7WT_MA	WT	1	1	1	1	1	1	1	1	1	1	1	1	1	1	1	1	1	1	1	1	1	1	1	1	1	1	1	1	1	1	1	1	0	0	0	0	
YJM311-8WT_MA	WT	1	1	1	1	1	1	1	1	1	1	1	1	1	1	1	1	1	1	1	1	1	1	1	1	1	1	1	1	1	1	1	1	0	0	0	0	
YJM311-9WT_MA	WT	1	1	1	1	1	1	1	1	1	1	1	1	1	1	1	1	1	1	1	1	1	1	1	1	1	1	1	1	1	1	1	1	0	0	1	1	LOH Chr7:621299-Rtel (Chr7b)
26_MA	complex	1	1	1	1	1	1	1	1	1	1	1	1	1	1	1	1	1	1	1	1	1	1	1	1	1	2	1	1	1	1	1	1	1	0	0	0	
18_MA	smooth	1	1	1	1	1	0	1	1	1	1	1	1	1	1	1	1	1	1	1	1	1	1	1	1	1	1	1	1	1	1	1	1	1	0	0	0	
98_MA	smooth	1	1	1	1	1	1	1	1	1	1	1	1	1	1	1	0	1	1	1	1	1	1	1	1	1	1	1	1	1	1	1	1	1	0	0	0	
99_MA	smooth	1	1	1	1	1	1	1	1	1	1	1	1	1	1	1	1	0	1	1	1	1	1	1	1	1	1	1	1	1	1	1	1	1	0	0	0	
97_MA	smooth	1	0	1	1	1	1	1	1	1	0	1	1	1	1	1	1	1	1	1	1	1	1	1	1	1	1	1	1	1	1	1	1	1	0	0	0	
37_MA	complex	1	0	1	1	1	1	1	1	1	1	1	1	1	1	1	1	1	1	1	1	1	1	1	1	1	1	1	1	1	1	1	1	1	1	1	2	LOH Chr13:227708-280885 (Chr13b)
6_MA	smooth	1	1	1	1	1	1	1	1	2	0	1	1	1	1	1	1	1	1	1	1	1	1	1	1	1	1	1	1	1	1	1	1	1	1	0	1	
33_MA	smooth	1	1	1	1	1	1	1	1	0	2	1	1	1	1	1	1	1	1	1	1	1	1	1	1	1	1	1	1	1	1	1	1	1	1	0	1	
47_MA	complex	1	1	1	1	1	1	1	1	1	1	1	1	1	2	1	1	1	1	2	1	1	1	1	1	1	1	1	1	1	1	1	1	2	0	0	0	
23_MA	complex	1	0	1	1	1	1	1	1	1	1	1	1	1	1	1	1	1	1	1	2	1	1	1	1	1	2	1	1	1	1	1	1	2	0	0	0	
15_MA	smooth	1	1	1	1	1	1	1	1	1	1	1	1	1	1	1	1	1	1	2	1	1	1	1	1	1	1	1	1	1	2	1	1	2	0	0	0	
17_MA	smooth	1	1	1	1	1	1	2	1	1	1	1	1	2	1	1	1	1	1	1	1	1	1	1	1	1	1	1	1	1	1	1	1	2	0	0	0	
30_MA	smooth	1	1	1	1	1	1	1	2	1	1	1	1	1	1	1	1	0	1	1	1	1	1	1	1	1	1	1	1	1	1	1	1	2	0	0	0	
102_MA	smooth	1	1	1	1	1	1	2	1	1	1	1	1	2	1	1	1	1	1	1	1	1	1	1	1	1	1	1	1	1	1	1	1	2	0	0	0	
24_MA	complex	1	1	1	1	1	1	2	1	1	1	1	1	1	1	1	1	1	1	1	1	1	1	1	1	2	1	1	1	1	1	1	1	2	0	0	0	
42_MA	complex	1	1	1	1	1	1	1	1	1	1	1	1	1	1	1	1	1	1	1	1	1	1	1	1	2	1	1	1	1	1	2	1	3	0	0	0	
44_MA	smooth	1	1	2	1	1	1	1	1	1	1	1	1	1	1	1	1	2	1	1	1	1	1	1	1	1	1	1	1	1	2	1	1	3	0	0	0	
41_MA	complex	1	2	2	1	1	1	1	1	1	1	1	1	1	1	1	1	1	1	1	1	1	1	1	1	2	1	1	1	1	1	1	2	4	0	0	0	
29_MA	smooth	1	1	1	1	1	1	1	2	1	1	1	1	1	2	1	1	1	1	2	2	1	1	1	1	1	1	1	1	1	2	1	1	5	0	0	0	
34_MA	smooth	1	1	2	1	1	1	1	2	1	1	1	1	1	1	1	1	1	2	1	2	1	1	1	1	1	1	1	2	1	1	2	1	6	0	0	0	
28_MA	complex	0	1	1	1	1	1	1	1	1	1	1	1	1	1	1	1	1	1	2	1	1	1	1	1	1	1	1	1	1	1	1	1	2	0	1	1	LOH Chr11:493199-489617 (Chr11b)
2_MA	smooth	1	0	1	1	1	1	1	1	1	1	1	1	1	1	1	1	1	1	1	1	1	1	1	1	1	1	1	1	2	1	1	1	2	0	1	1	LOH Chr15: 701405-Rtel (Chr15a)
4_MA	smooth	1	0	1	1	1	1	1	1	1	1	1	1	1	1	1	1	1	1	0	2	1	1	1	1	1	1	1	1	1	1	1	1	2	0	1	1	LOH Chr15: Ltel-185281 (Chr15a)
5_MA	smooth	1	1	1	1	1	1	2	2	1	1	1	1	1	1	1	1	1	1	1	2	1	1	1	2	2	2	1	1	1	1	1	1	6	0	1	1	LOH Chr4:1,434,135-Rtel (Chr4b)
32_MA	smooth	1	1	1	1	1	1	1	1	1	1	1	2	1	1	1	1	1	1	1	1	1	1	2	1	1	2	1	1	1	2	1	1	3	1	0	1	
20_MA	smooth	1	1	2	0	1	2	1	1	1	1	1	2	2	1	1	1	2	1	1	2	1	2	2	1	2	1	2	1	2	2	2	2	16	1	0	1	
52_MA	smooth	1	1	1	2	1	1	1	1	1	1	1	2	1	1	1	1	2	1	1	1	1	1	1	2	1	1	1	1	1	2	1	1	6	1	1	2	LOH Chr16:593641-Rtel (Chr16a)
49_MA	complex	1	1	1	1	2	1	1	1	1	1	1	1	1	1	2	1	1	1	1	1	1	1	1	1	1	1	1	1	1	1	1	1	3	1	2	3	LOH Chr12:319640-442777 (Chr12a), LOH Chr13:66323-69300 (chr13b)
25_MA	complex	1	1	1	1	1	1	2	1	1	1	1	2	1	1	1	1	1	2	1	1	1	1	1	1	1	2	1	1	2	1	1	1	4	2	0	2	
40_MA	complex	2	0	1	2	1	1	1	1	1	1	1	1	1	2	2	1	2	0	2	1	1	1	1	1	1	1	1	1	1	1	1	1	8	3	0	3	
36_MA	complex	1	0	1	2	2	1	1	2	1	1	2	0	1	1	1	2	2	0	2	0	2	1	2	1	2	2	1	2	2	0	0	2	3	14	0	14	
