# Supplementary figures and images for: Bursts of Genomic Instability Potentiate Phenotypic and Genomic Diversification in Saccharomyces cerevisiae
Source: Front Genet. 2022 Jun 17;13:912851. doi: 10.3389/fgene.2022.912851 (PMC9247159; doi:10.3389/fgene.2022.912851)

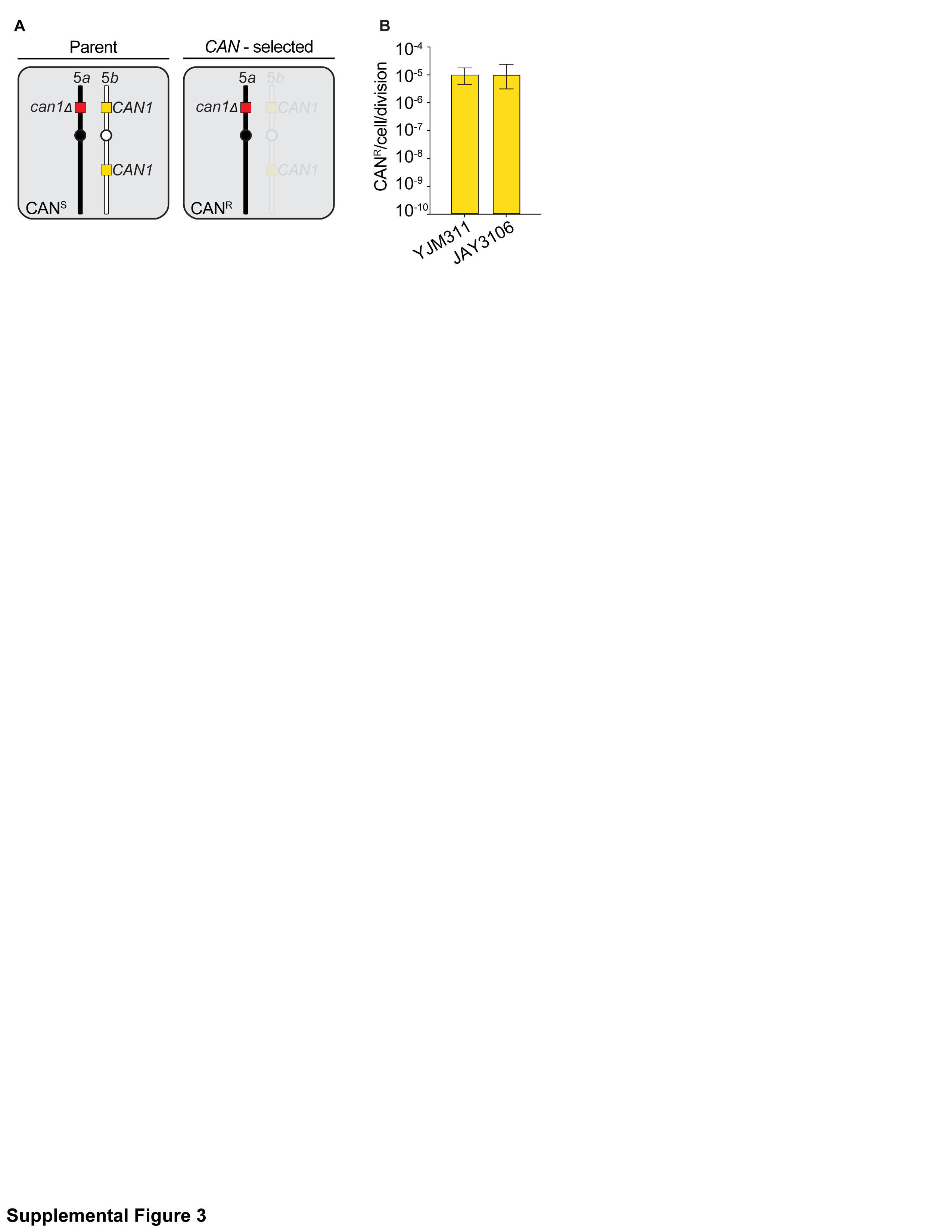

Supplement: Supplementary file 3 [file Image3.tif]

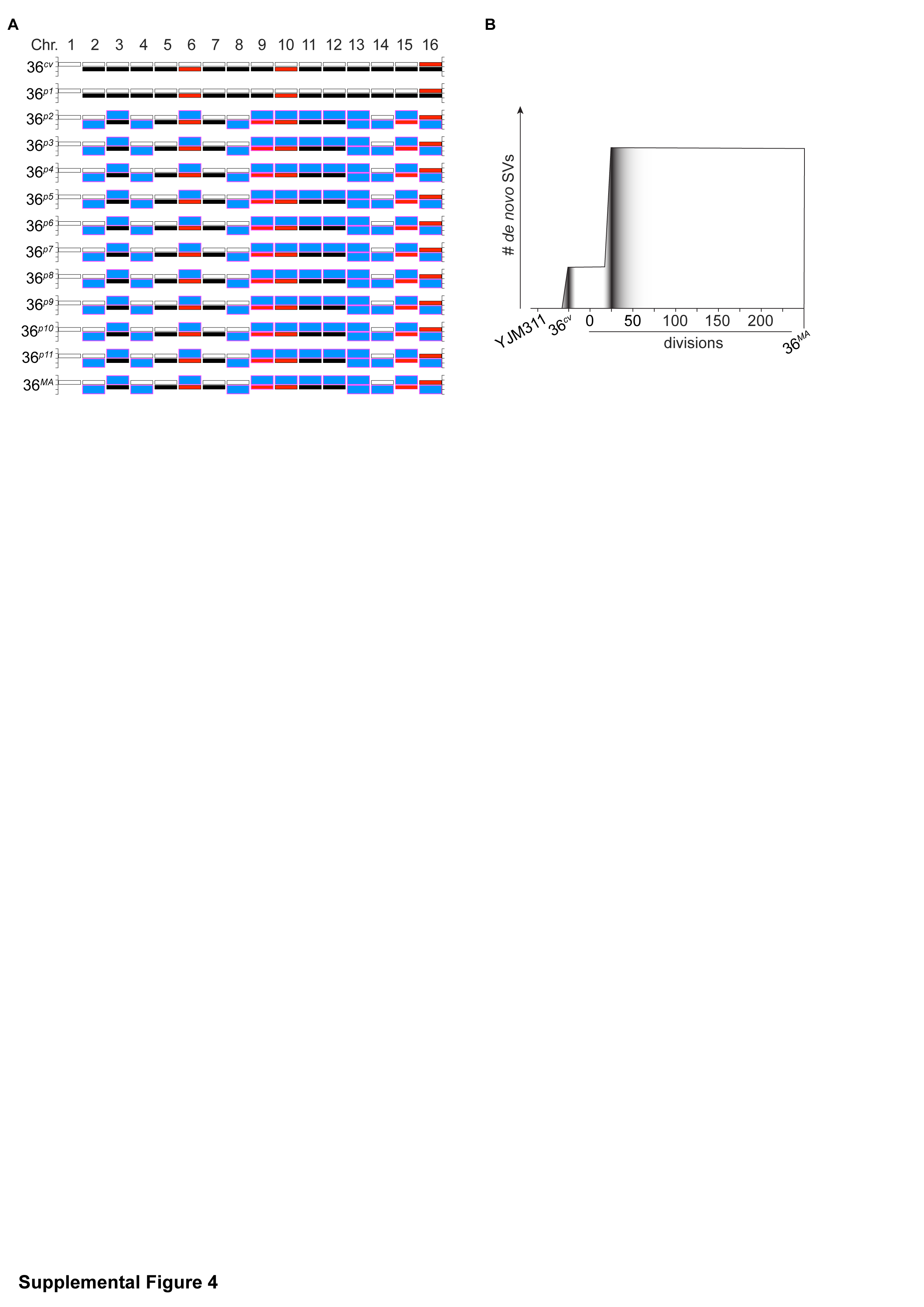

Supplement: Supplementary file 4 [file Image4.tif]

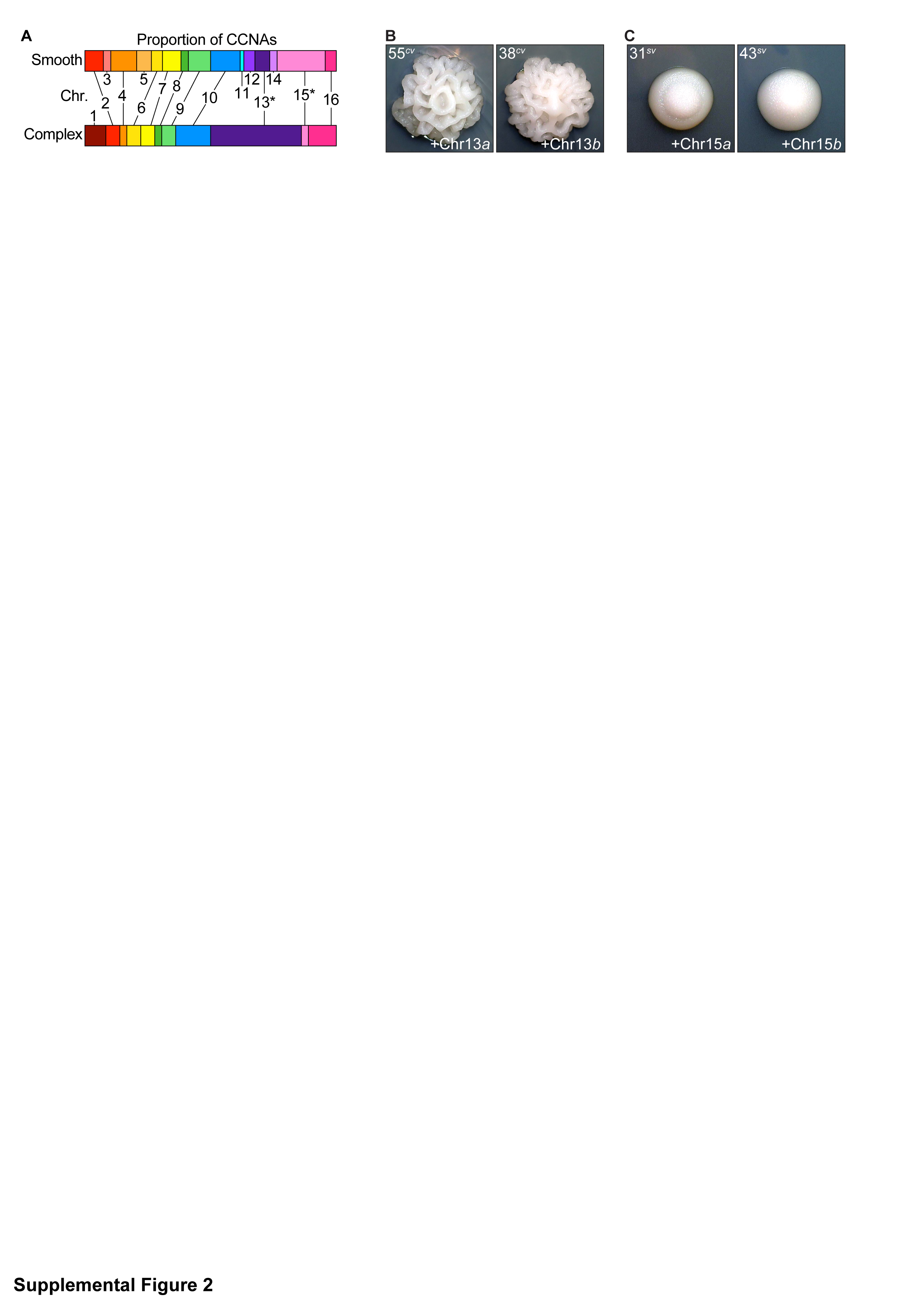

Supplement: Supplementary file 5 [file Image2.tif]

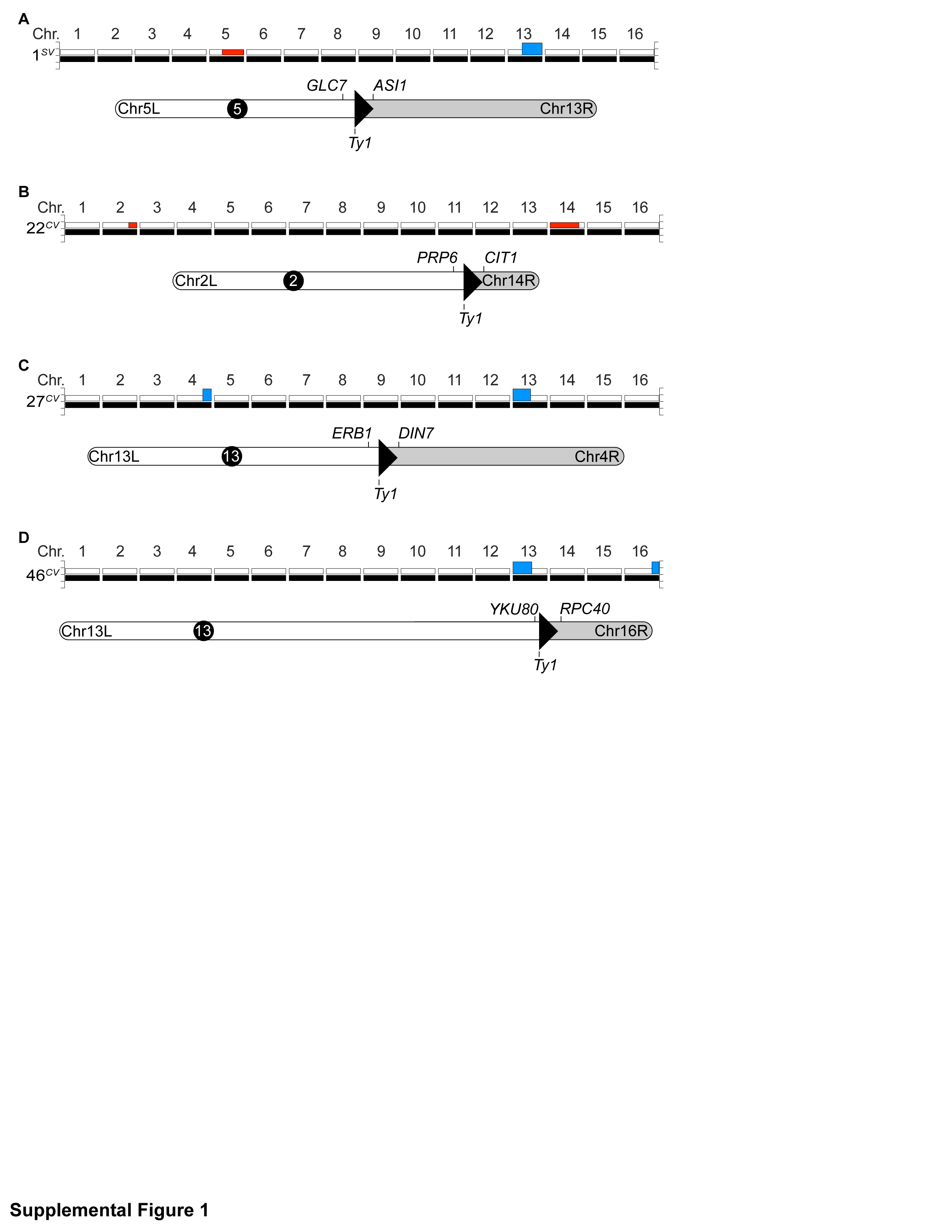

Supplement: Supplementary file 6 [file Image1.tif]
