# Supplementary material for: Bursts of Genomic Instability Potentiate Phenotypic and Genomic Diversification in Saccharomyces cerevisiae
Source: Front Genet. 2022 Jun 17;13:912851. doi: 10.3389/fgene.2022.912851 (PMC9247159; doi:10.3389/fgene.2022.912851)
Supplement: Supplementary file 7 [file DataSheet3.PDF]

Table S3. Fluctuation analysis derived rates of Chr5 loss in YJM311 background				
Selection	Rate	Upper 95% difference	Lower 95% difference	Number of cultures
YJM311	1.04E-05	7.43E-06	5.80E-06	24
JAY3106	1.03E-05	1.39E-05	7.14E-06	12
