# Supplementary material for: Bursts of Genomic Instability Potentiate Phenotypic and Genomic Diversification in Saccharomyces cerevisiae
Source: Front Genet. 2022 Jun 17;13:912851. doi: 10.3389/fgene.2022.912851 (PMC9247159; doi:10.3389/fgene.2022.912851)
Supplement: Supplementary file 8 [file DataSheet1.PDF]

Table S1. Copy Number Analysis of Chr1 in YJM311 and WT derivative clones. For comparison, copy number analysis for denoted region of Chr7 is also shown. Blue cells highlight WT clones that are monosomic for Chr1.

	genomic region: chr1:50,000-150,000; 427 hetSNPs		genomic region: chr7:200,000-300,000; 66 hetSNPs	
**Isolate**	**log2 coverage (median)**	**Copy Number**	**log2 coverage (median)**	**Copy Number**
YJM311	-0.18	1.76	-0.02	1.97
57	-0.07	1.91	-0.02	1.97
58	-0.03	1.96	-0.01	1.98
59	-0.24	1.69	-0.02	1.97
60	-0.04	1.95	-0.03	1.95
61	-0.07	1.91	-0.03	1.96
62	-0.08	1.89	-0.02	1.97
63	-0.85	1.11	-0.02	1.97
64	-0.88	1.09	-0.03	1.95
65	-0.04	1.94	-0.01	1.98
66	-0.84	1.11	-0.04	1.95
77	-0.09	1.88	-0.03	1.96
78	-0.12	1.84	-0.01	1.98
79	-0.04	1.94	-0.02	1.97
80	-0.08	1.89	-0.02	1.97
81	-0.88	1.09	-0.03	1.96
82	-0.17	1.77	-0.02	1.98
83	-0.05	1.93	-0.01	1.98
84	-0.05	1.93	-0.03	1.96
85	-0.14	1.82	-0.01	1.98
86	-0.05	1.93	-0.01	1.99
